# Supplementary material for: RNA virus interference via CRISPR/Cas13a system in plants
Source: Genome Biol. 2018 Jan 4;19:1. doi: 10.1186/s13059-017-1381-1 (PMC5755456; doi:10.1186/s13059-017-1381-1)
Supplement: Supplementary file 1 — Confirmation of pCas13a expression in planta. Figure S2. pCas13a-mediated interference with TuMV-GFP in planta. Figure S3. GFP quantification of TuMV-GFP interference in transgenic pCas13a-OE plants. (PDF 1393 kb) [file 13059_2017_1381_MOESM1_ESM.pdf]

ADDITIONAL FILE 1

RNA virus interference via CRISPR/Cas13a system in plants

Rashid Aman[1, 3], Zahir Ali[1, 3], Haroon Butt[1, 3], Ahmed Mahas[1], Fatimah Aljedaani[1], Muhammad Zuhaib Khan[1], Shouwei Ding[2], and Magdy Mahfouz[1, *]

[1]Laboratory for Genome Engineering, Division of Biological Sciences, 4700 King Abdullah University of Science and Technology, Thuwal 23955-6900, Saudi Arabia, and [2]Center for Plant Cell Biology, Department of Microbiology and Plant Pathology, University of California, Riverside, CA 92521

***Corresponding author:** Magdy M. Mahfouz (magdy.mahfouz@kaust.edu.sa)

Additional file 1

Figure S1: Confirmation of *pCas13a* expression in *planta*.

Figure S2: pCas13a-mediated interference with TuMV-GFP in *planta*.

Figure S3: GFP quantification of TuMV-GFP interference in transgenic pCas13a-OE plants.

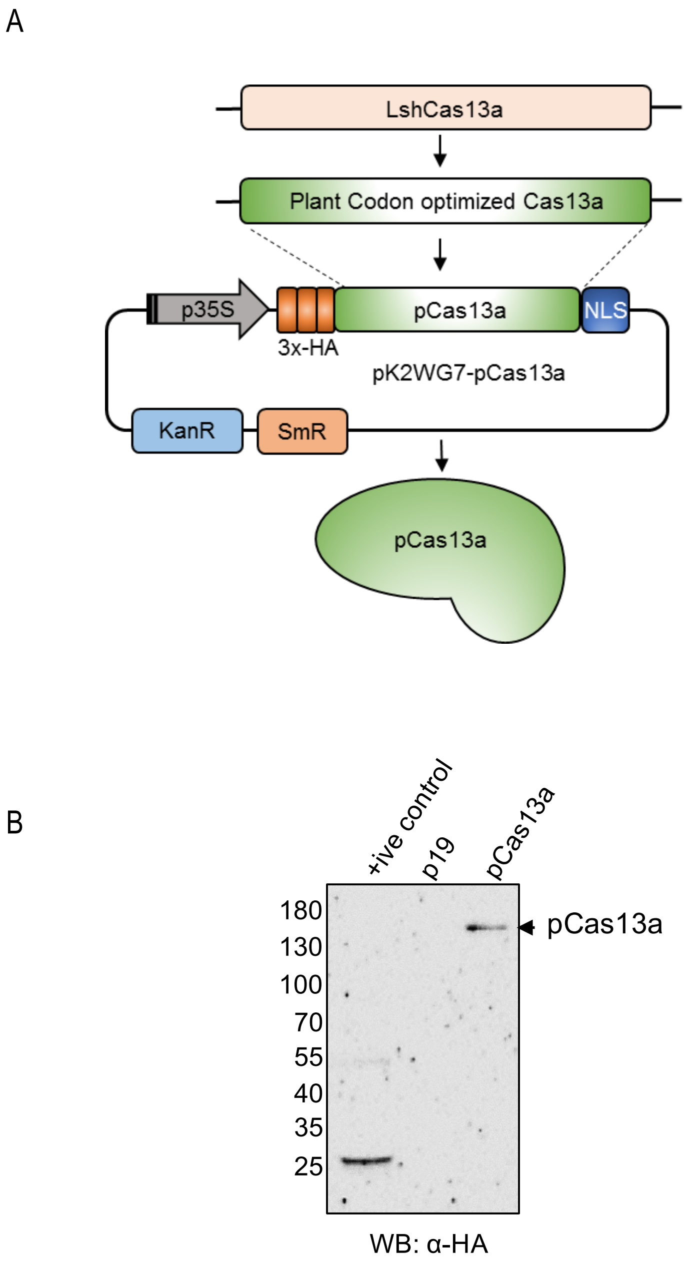

Figure S1. Confirmation of *pCas13a* expression in *planta*.

A) Schematic for the expression of Cas13a in *planta*. The *LshCas13a* was plant codon optimized (pCas13a) and cloned into the binary vector *pK2GW7*.

B) Proteins were extracted from *N. benthamiana* leaves infiltrated with Agrobacterium having the binary construct *pK2GW7-pCas13a* and p19. The protein blot was developed for the detection of HA-pCas13a with anti-HA antibody. The *N. benthamiana* line expressing an unrelated 27-kDa HA-

tagged protein was used as positive control. P19 alone was used as the negative control. The arrow indicates the detection of the expected size 171 kDa pCas13a.

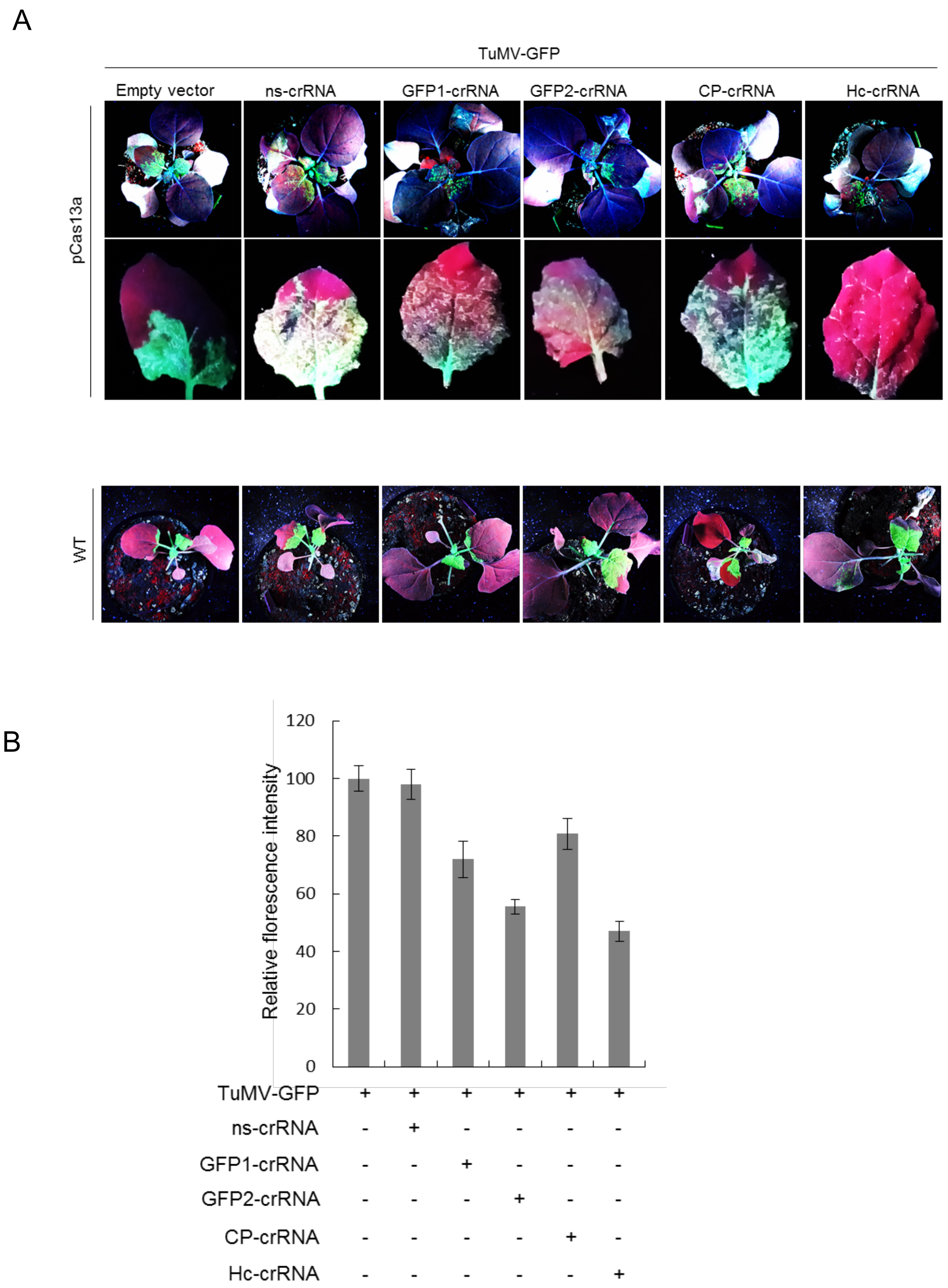

Figure S2. pCas13a-mediated interference with TuMV-GFP in *planta*.

A) GFP imaging of the viral interference in plants transiently expressing pCas13a and crRNA. Agrobacterium containing the *pK2GW7-pCas13a*, TuMV-GFP and TRV expressing crRNA (against GFP, HC-pro and CP of TuMV-GFP) were infiltrated into wild-type *N. benthamiana*.

Plants were imaged under UV-light for the systemic spread of TuMV-GFP at 7 dai. The representative third leaf of individual plants are shown in the middle panel. The lower panel represents the WT *N. benthamiana* infiltrated with TuMV-GFP and TRV expressing the respective crRNAs without pCas13a.

B) Relative interference of TuMV-GFP. The GFP intensity was used as relative reference to measure the pCas13a-mediated interference of TuMV-GFP. GFP signal was quantified for the reduction of the TuMV-GFP in plants (*n*=5).

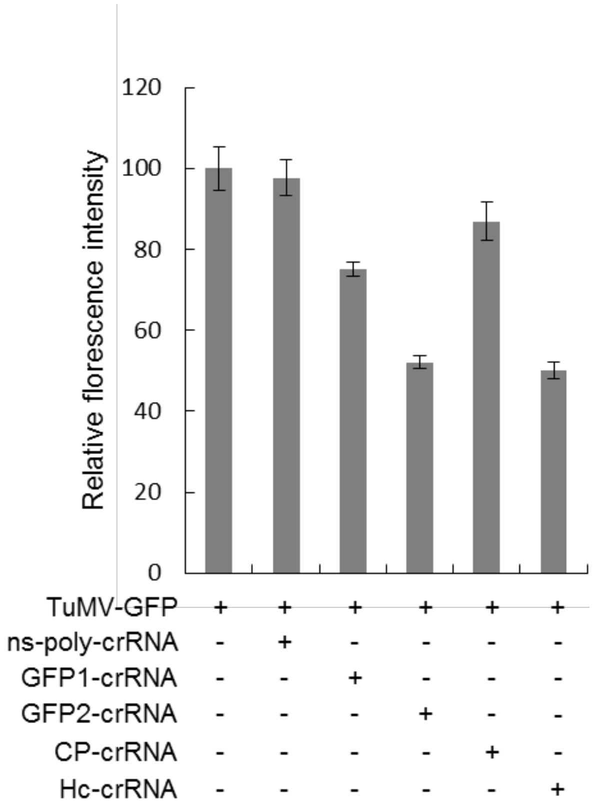

Figure S3. GFP quantification of TuMV-GFP interference in transgenic pCas13a-OE plants.

The GFP signal (plants *n*=5) was used for the generation of graph representing the comparative reduction of the GFP signal upon targeting.
