# Supplementary material for: RNA virus interference via CRISPR/Cas13a system in plants
Source: Genome Biol. 2018 Jan 4;19:1. doi: 10.1186/s13059-017-1381-1 (PMC5755456; doi:10.1186/s13059-017-1381-1)
Supplement: Supplementary file 2 — pCas13a amino acid sequence (3xHA-pCas13a-nls). Sequence S2: pCas13a full-length plant codon optimized DNA sequence (3x-HA-pCas13a-nls) Map S1: pCas13a sequence in pK2GW7-pCas13a (pK2GW7-3xHA-pCas13a-nls). Sequence S3: TuMV-GFP full-length sequence with target sequences in different colors. Map S2: Map of TuMV-GFP. Sequence S4: Cas13a-repeat-cRNA-TuMV-GFP-GFP-target 1 sequence (A), map (B) and its complex with targeting region in TuMV GFP genomic region (C). Sequence S5: Cas13a-repeat-cRNA-TuMV-GFP-GFP-T2 sequence (A), map (B), and its complex with targeting area in TuMV GFP genomic region (C). Sequence S6: Cas13a-repeat-cRNA-TuMV-GFP-HC-Pro-T1 sequence (A), map (B), and its complex with HC-pro targeting region in TuMV (C). Sequence S7: Cas13a-repeat-cRNA-TuMV-GFP-Cp-Pro-T1 sequence (A), map (B), and its complex with Capsid protein targeting region in TuMV. (DOCX 414 kb) [file 13059_2017_1381_MOESM2_ESM.docx]

**ADDITIONAL FILE 2**

**RNA virus interference via CRISPR/Cas13a system in plants**

Rashid Aman^1, 3^, Zahir Ali^1, 3^, Haroon Butt^1, 3^, Ahmed Mahas^1^, Fatimah Aljedaani^1^, Muhammad

Zuhaib Khan^1^, Shouwei Ding^2^, and Magdy Mahfouz^1, *^

^1^Laboratory for Genome Engineering, Division of Biological Sciences, 4700 King Abdullah University of Science and Technology, Thuwal 23955-6900, Saudi Arabia, and ^2^Center for Plant Cell Biology, Department of Microbiology and Plant Pathology, University of California, Riverside, CA 92521

***Corresponding author:** Magdy M. Mahfouz ([magdy.mahfouz@kaust.edu.sa)](mailto:magdy.mahfouz@kaust.edu.sa))

**Additional file 2.**

**Sequence S1:** **pCas13a amino acid sequence (3xHA-pCas13a-nls)**

**Sequence S2: pCas13a full-length plant codon optimized DNA sequence (3x-HA-pCas13a-nls)**

**Map S1: pCas13a sequence in pK2GW7-pCas13a (pK2GW7-3xHA-pCas13a-nls)**

**Sequence S3: TuMV-GFP full length sequence with target sequences in different colors**

**Map S2:** **Map of TuMV-GFP**

**Sequence S4: Cas13a-repeat-cRNA-TuMV-GFP-GFP-target 1 sequence (A), map (B) and its complex with targeting region in TuMV GFP genomic region (C)**

**Sequence S5: Cas13a-repeat-cRNA-TuMV-GFP-GFP-T2 sequence (A), map (B) and its complex with targeting area in TuMV GFP genomic region (C).**

**Sequence S6: Cas13a-repeat-cRNA-TuMV-GFP-HC-Pro-T1 sequence (A), map (B) and its complex with HC-pro targeting region in TuMV (C).**

**Sequence S7: Cas13a-repeat-cRNA-TuMV-GFP-Cp-Pro-T1 sequence (A), map (B) and its complex with Capsid protein targeting region in TuMV.**

**Sequence S1:** pCas13a amino acid sequence (3xHA-pCas13a-nls)

(3x-HA and NLS are highlighted)

MYPYDVPDYAYPYDVPDYAYPYDVPDYAGNLFGHKRWYEVRDKKDFKIKRKVKVKRNYDGNKYILNINENNNKEKIDNNKFIRKYINYKKNDNILKEFTRKFHAGNILFKLKGKEGIIRIENNDDFLETEEVVLYIEAYGKSEKLKALGITKKKIIDEAIRQGITKDDKKIEIKRQENEEEIEIDIRDEYTNKTLNDCSIILRIIENDELETKKSIYEIFKNINMSLYKIIEKIIENETEKVFENRYYEEHLREKLLKDDKIDVILTNFMEIREKIKSNLEILGFVKFYLNVGGDKKKSKNKKMLVEKILNINVDLTVEDIADFVIKELEFWNITKRIEKVKKVNNEFLEKRRNRTYIKSYVLLDKHEKFKIERENKKDKIVKFFVENIKNNSIKEKIEKILAEFKIDELIKKLEKELKKGNCDTEIFGIFKKHYKVNFDSKKFSKKSDEEKELYKIIYRYLKGRIEKILVNEQKVRLKKMEKIEIEKILNESILSEKILKRVKQYTLEHIMYLGKLRHNDIDMTTVNTDDFSRLHAKEELDLELITFFASTNMELNKIFSRENINNDENIDFFGGDREKNYVLDKKILNSKIKIIRDLDFIDNKNNITNNFIRKFTKIGTNERNRILHAISKERDLQGTQDDYNKVINIIQNLKISDEEVSKALNLDVVFKDKKNIITKINDIKISEENNNDIKYLPSFSKVLPEILNLYRNNPKNEPFDTIETEKIVLNALIYVNKELYKKLILEDDLEENESKNIFLQELKKTLGNIDEIDENIIENYYKNAQISASKGNNKAIKKYQKKVIECYIGYLRKNYEELFDFSDFKMNIQEIKKQIKDINDNKTYERITVKTSDKTIVINDDFEYIISIFALLNSNAVINKIRNRFFATSVWLNTSEYQNIIDILDEIMQLNTLRNECITENWNLNLEEFIQKMKEIEKDFDDFKIQTKKEIFNNYYEDIKNNILTEFKDDINGCDVLEKKLEKIVIFDDETKFEIDKKSNILQDEQRKLSNINKKDLKKKVDQYIKDKDQEIKSKILCRIIFNSDFLKKYKKEIDNLIEDMESENENKFQEIYYPKERKNELYIYKKNLFLNIGNPNFDKIYGLISNDIKMADAKFLFNIDGKNIRKNKISEIDAILKNLNDKLNGYSKEYKEKYIKKLKENDDFFAKNIQNKNYKSFEKDYNRVSEYKKIRDLVEFNYLNKIESYLIDINWKLAIQMARFERDMHYIVNGLRELGIIKLSGYNTGISRAYPKRNGSDGFYTTTAYYKFFDEESYKKFEKICYGFGIDLSENSEINKPENESIRNYISHFYIVRNPFADYSIAEQIDRVSNLLSYSTRYNNSTYASVFEVFKKDVNLDYDELKKKFKLIGNNDILERLMKPKKVSVLELESYNSDYIKNLIIELLTKIENTNDTLKRPAATKKAGQAKKKK*

**Sequence S2:** pCas13a full-length plant codon optimized DNA sequence (3x-HA-pCas13a-nls)

ATGTACCCATACGATGTTCCAGATTACGCTTACCCATACGATGTTCCAGATTACGCTTACCCATACGATGTTCCAGATTACGCTGGCAACCTCTTTGGCCATAAGCGTTGGTACGAAGTCAGAGATAAGAAGGACTTCAAGATTAAGAGAAAGGTCAAGGTGAAGCGCAACTACGACGGAAACAAGTACATCTTGAACATAAACGAAAACAACAACAAGGAAAAGATCGATAACAACAAGTTTATTCGTAAGTACATCAACTATAAGAAGAACGACAACATACTAAAGGAGTTTACAAGGAAGTTTCATGCCGGTAACATTCTCTTCAAGTTGAAAGGAAAGGAGGGCATCATCCGTATCGAAAACAACGACGATTTCCTCGAGACTGAAGAAGTGGTCCTTTACATTGAGGCTTACGGTAAAAGCGAGAAGCTAAAGGCCCTCGGAATCACCAAGAAGAAGATCATCGATGAAGCGATAAGACAGGGTATCACTAAGGATGACAAGAAGATCGAGATCAAGCGTCAGGAAAACGAGGAGGAGATCGAGATAGACATTCGTGATGAGTATACTAACAAGACCCTAAACGACTGCTCCATCATCCTCCGTATTATCGAAAACGATGAGCTCGAAACAAAAAAGAGCATTTACGAGATTTTCAAGAACATTAACATGAGTCTCTACAAGATCATTGAGAAGATCATAGAAAACGAGACAGAAAAAGTTTTCGAGAACAGATACTACGAGGAGCATTTGCGTGAAAAGCTCCTCAAAGATGACAAGATAGATGTGATCTTGACTAACTTCATGGAAATTAGGGAAAAGATCAAAAGTAACCTCGAGATTTTGGGCTTCGTCAAGTTCTACCTCAACGTTGGAGGTGACAAGAAGAAGTCTAAGAACAAGAAAATGTTGGTCGAAAAGATCCTAAACATCAACGTGGATTTGACAGTGGAGGACATAGCCGACTTCGTGATTAAGGAACTCGAGTTCTGGAACATCACCAAGCGTATCGAAAAGGTGAAGAAGGTGAACAACGAATTCCTAGAAAAGAGGAGGAACCGTACATATATAAAGTCTTACGTGCTTCTCGACAAGCACGAAAAGTTCAAGATTGAGCGTGAGAACAAGAAGGACAAGATCGTGAAGTTTTTCGTTGAGAACATCAAGAACAACTCGATCAAGGAAAAGATAGAGAAGATCCTCGCGGAATTTAAGATTGACGAATTGATAAAAAAGCTAGAGAAGGAGCTCAAGAAGGGCAACTGCGATACCGAAATTTTCGGTATATTCAAGAAGCACTATAAGGTTAACTTCGACAGTAAGAAATTCTCTAAGAAATCTGACGAGGAGAAGGAACTATATAAAATTATCTACCGTTACCTCAAGGGCAGAATCGAGAAAATCTTGGTCAACGAGCAGAAGGTGCGTCTTAAGAAGATGGAAAAGATCGAAATTGAAAAAATCTTGAACGAGTCTATTCTTTCTGAAAAGATCCTAAAAAGAGTGAAGCAGTACACTCTAGAACACATCATGTACTTGGGCAAGCTACGTCACAACGACATCGACATGACTACCGTTAACACCGACGACTTCTCTAGGCTCCACGCCAAAGAGGAGCTAGACCTAGAGCTCATTACATTCTTCGCCTCGACAAACATGGAGCTCAACAAGATCTTTTCTCGTGAAAACATCAACAACGACGAGAACATTGACTTCTTCGGTGGAGACAGAGAAAAAAACTACGTTCTCGACAAGAAAATCCTCAACTCTAAGATCAAAATAATACGTGACTTGGACTTCATCGACAACAAAAACAACATAACAAACAACTTCATTCGTAAGTTTACAAAGATCGGTACCAACGAGCGTAACCGCATCTTGCATGCTATCTCCAAGGAGCGTGACCTACAGGGTACCCAGGATGACTACAACAAGGTCATCAACATCATCCAGAACCTCAAGATTTCCGATGAAGAAGTGTCTAAGGCTTTGAACTTGGACGTCGTCTTCAAGGACAAGAAAAACATAATAACTAAAATCAACGATATAAAGATATCTGAGGAAAACAACAACGATATCAAGTATCTCCCTTCCTTCAGTAAGGTTCTCCCAGAGATCCTCAACCTCTATAGAAACAACCCCAAGAACGAACCTTTTGACACAATTGAGACAGAAAAGATCGTCCTCAACGCACTTATCTACGTTAACAAAGAGTTGTACAAGAAGTTGATCCTCGAGGACGATTTGGAAGAGAACGAGAGTAAGAACATATTCCTTCAGGAGCTTAAGAAGACCCTAGGTAACATTGACGAAATCGATGAAAACATAATAGAAAACTACTATAAGAACGCCCAGATCAGCGCGTCCAAGGGTAACAACAAGGCGATCAAGAAGTACCAGAAGAAGGTGATCGAGTGCTACATAGGTTACCTCCGTAAGAACTACGAGGAACTCTTTGACTTCTCTGATTTTAAGATGAACATTCAGGAGATCAAGAAGCAGATTAAGGACATTAACGATAACAAGACTTACGAAAGGATCACAGTGAAGACAAGCGATAAGACCATAGTGATCAACGACGACTTCGAGTATATAATATCTATCTTTGCCCTCCTCAACTCGAACGCCGTGATCAACAAGATCAGGAACAGATTCTTTGCCACATCGGTCTGGCTTAACACCAGCGAGTATCAGAACATCATTGACATCTTGGACGAAATAATGCAGTTGAACACTTTGCGCAACGAGTGTATCACTGAGAACTGGAACCTCAACCTCGAGGAGTTCATACAGAAGATGAAGGAAATTGAGAAGGACTTCGATGACTTCAAGATCCAGACCAAGAAGGAGATCTTCAACAACTACTACGAGGATATCAAGAACAACATTCTTACAGAGTTCAAGGACGATATCAACGGTTGCGACGTCCTTGAAAAAAAGTTGGAAAAAATTGTCATTTTTGACGATGAAACTAAATTTGAAATTGATAAGAAATCTAACATACTCCAGGACGAACAGAGGAAGCTCTCCAACATCAACAAGAAGGACCTCAAGAAGAAGGTGGACCAGTACATCAAGGACAAGGACCAGGAAATCAAGAGCAAGATCTTGTGCAGAATCATCTTCAACTCTGATTTCCTCAAAAAGTACAAGAAAGAAATCGATAACCTAATAGAGGATATGGAGAGTGAAAACGAGAACAAGTTCCAGGAGATCTACTATCCTAAGGAGAGAAAGAACGAGCTCTACATATATAAAAAGAACCTCTTCCTCAACATCGGTAACCCTAACTTCGATAAGATCTACGGTTTGATCTCCAACGACATAAAAATGGCTGACGCCAAGTTTCTCTTCAACATAGACGGCAAGAACATCAGGAAGAACAAGATATCCGAGATCGACGCCATCCTCAAGAACCTCAACGACAAGCTCAACGGATACAGTAAGGAATACAAGGAGAAGTACATCAAGAAACTTAAGGAAAACGACGATTTCTTTGCTAAGAACATCCAGAACAAGAACTACAAGTCTTTCGAAAAGGACTACAACCGTGTCAGCGAATATAAGAAGATCCGTGATCTTGTTGAATTCAACTATCTCAACAAGATCGAAAGTTACCTCATCGACATTAACTGGAAACTCGCTATCCAGATGGCGCGTTTCGAAAGGGACATGCATTATATCGTGAACGGACTCCGCGAGCTCGGCATAATCAAACTCTCCGGTTACAACACTGGCATCTCGCGTGCCTACCCAAAGCGTAACGGTAGCGACGGCTTCTACACCACCACCGCTTACTACAAGTTCTTCGATGAAGAGTCTTACAAGAAGTTCGAAAAAATCTGCTACGGCTTCGGAATCGACCTATCTGAGAACTCTGAGATTAACAAACCTGAGAACGAGAGCATCCGTAACTATATCTCCCATTTTTACATCGTGAGAAACCCTTTTGCTGACTACTCCATCGCTGAACAGATAGATCGTGTCTCTAACTTGTTGTCGTATTCCACCCGTTATAACAACAGTACTTATGCATCGGTGTTCGAAGTGTTCAAGAAAGACGTCAACTTGGACTACGACGAGCTCAAGAAAAAGTTCAAACTAATAGGTAACAACGATATCCTCGAGCGTTTGATGAAGCCTAAAAAAGTCTCTGTGCTCGAGCTCGAGTCTTACAACTCTGACTATATCAAGAACCTCATCATCGAGCTATTGACCAAGATCGAGAACACCAACGACACCCTTAAAAGGCCGGCGGCCACGAAAAAGGCCGGCCAGGCAAAAAAGAAAAAGTGA

**Map S1:** *pCas13a* sequence in *pK2GW7-pCas13a* *(pK2GW7-3xHA-pCas13a-nls)*

**
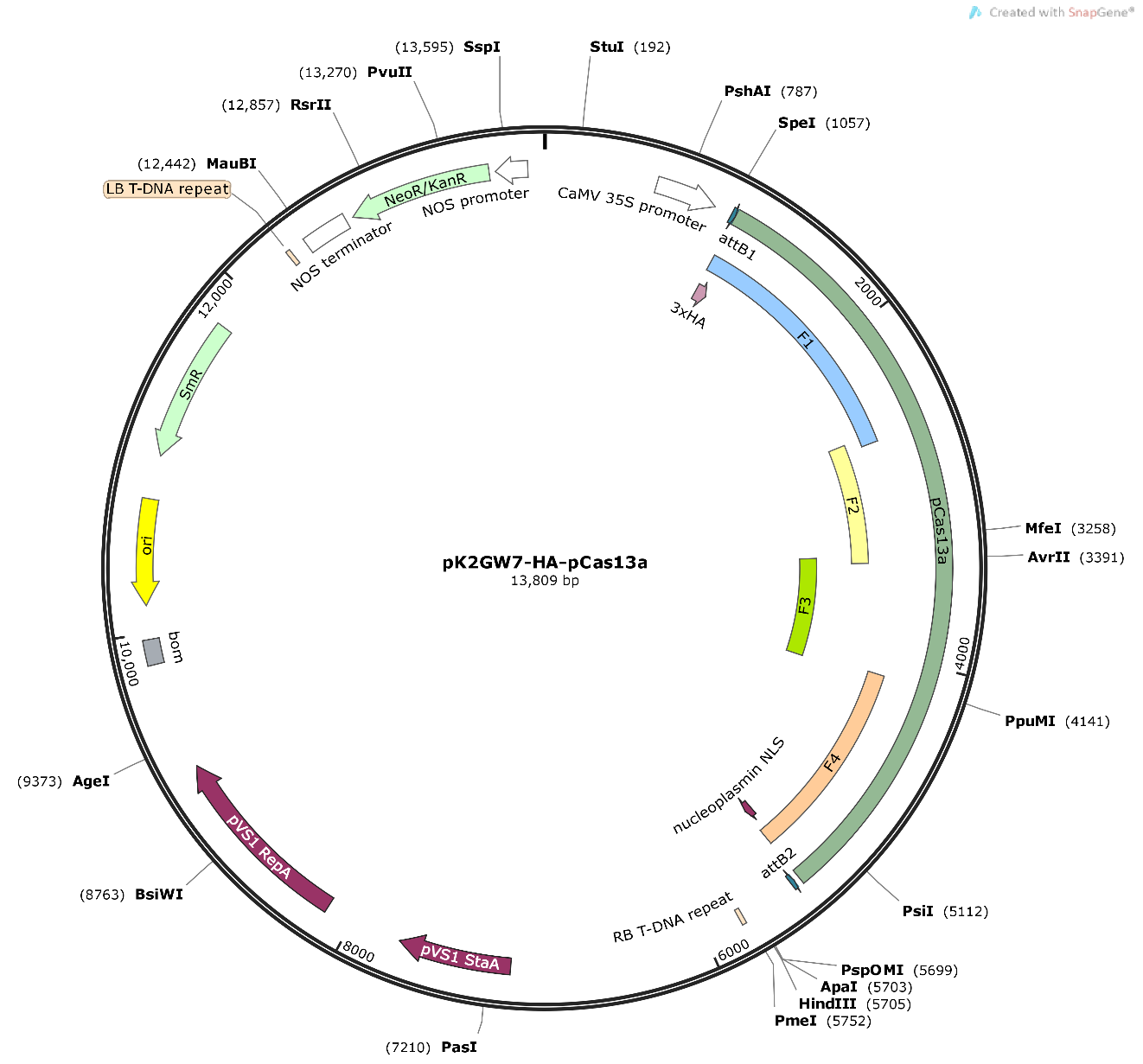
**

**Sequence S3:** *TuMV-GFP* full length sequence with target sequences in different colors

aaaaaatataaaaactcaacataacatacacaaaacgattaaagcaaacacaaatctttcaaagcattcaagcaatcaaagattctcaaatctttcatcgttatcaaagcaatcaccaacagcaaaccaaatggcagcagttacattcgcatcagctatcaccaacgccatcaccagcaaaccagcactcaccggaatggtgcagtttgggagtttcccaccaatgccattgcgatccaccaccgtcaccacagtcgccacttcagtggcgcaacctaaactgtacacagtgcagtttggaagccttgacccagtagtcgtcaagagtggagcagggtcccttgctaaggcaacacgccagcagcctaacgttgaaatagacgttagcctcagtgaagccgcagctctggaggttgcgaaacctagatcgaatgccgtgttgaggatgcacgaggaagcaaacaaggagagagcactctttttggactgggaggctagtttgaagagaagctcgtatggaattgctgaggacgagaaggttgtaatgacaactcatggcgtcagcaagatagtgcccagaagttcaagggcaatgaagctaaagcgcgcaagggagaggcgtagagcgcagcaaccaattatattaaagtgggagcccaaattgagcgggatctcaatcggaggagggctctctgcgagcgtaatcgaagcagaagaggttcgcacaaagtggccgcttcataagacaccgtcaatgaagaagaggacggtgcacagaatatgcaagatgaacgaccaaggagttgacatgttgacacgatccctggttaagattttcaagactaagagtgccaacattgaatacatcggaaagaagtcgattaaggtcgatttcatcagaaaagaacgaacgaaattcgcaagaatccaagtagcacacttactcgggaagagagcacagcgcgacttgttaactggaatggaagaaaaccattttattgacattctcagtaagtactcaggtaacaaaacaaccataaatcctggagtagtttgcgcaggttggagtggcatagtcgttggaaatggaattctaacccagaaacgaagcagaagtccatcagaggcctttgtaattagaggtgagcacgaaggcaagttgtacgatgccaggatcaaagtcacgaggacaatgagtcacaagattgtgcactttagtgccatggcaagtaaaggagaagaacttttcactggagttgtcccaattcttgttgaattagatggtgatgttaatgggcacaaattttctgtcagtggagagggtgaaggtgatgcaacatacggaaaacttaccc**ttaaatttatttgcactactggaaaactacctgttc**cttggccaacacttgtcactactttctcttatggtgttcaatgcttttcaagatacccagatcatatgaagcggcacgacttcttcaagagcgccatgcctgagggatacgtgcaggagaggaccatctctttcaaggacgacgggaactacaagacacgtgctgaagtcaagtttgagggagacaccctcgtcaacaggat**cgagcttaagggaatcgatttcaaggaggacgga**aacatcctcggccacaagttggaatacaactacaactcccacaacgtatacatcacggcagacaaacaaaagaatggaatcaaagctaacttcaaaattagacacaacattgaagatggaagcgttcaactagcagaccattatcaacaaaatactccaattggcgatggccctgtccttttaccagacaaccattacctgtccacacaatctgccctttcgaaagatcccaacgaaaagagagaccacatggtccttcttgagtttgtaacagctgctgggattacacatggcatggatgaactatacaaaggatccgttgaggcttgtgtctatcaccaggcaggtgcagcgggagccaacttctggaaaggcttcgacagatgctttctcgcataccgtagtgacaatcgcgagcatacatgctattcagggctagatgtcactgagtgcggcgaggtggcagcactgatgtgtttggctatgttcccatgcggaaagataacctgccctgactgtgtaa**cagatagtgagctatcccaaggacaagcaagcgg**accatctatgaagcacaggttgacacagctacgcgatgtcatcaagtcaagctacccacgcttcaagcatgcagtgcagatactagataggtatgagcaatcactgagcagtgcaaacgagaactaccaagatttcgcagaaatccagagcataagcgatggagttgaaaaagctgcattcccacacgtcaacaagctaaacgcaatattgatcaaaggggccacagtaacaggagaggaattctcgcaggctacgaagcacttgctcgagatagcacgatacctgaagaacagaaccgagaacattgagaagggttcactgaagtcctttcgcaacaagatttcccagaaagcgcacatcaacccaacactaatgtgtgacaaccagctcgatagaaatggaaatttcatatggggtgagagaggataccatgcaaaacgattcttcagcaactactttgaaataatcgatccaaagaaaggctacacccaatacgagacaagagcggtaccaaatgggtcacggaaacttgcaatcggcaaactaatagtcccaacgaacttcgaagttttaagggaacagatgaaaggcgaaccggtagaaccatacccagtaacagtcgagtgtgtgagcaagttacagggtgacttcgtccatgcatgttgttgtgtcacaacagaatcaggcgacccagtcttgtctgagatcaaaatgccaaccaaacaccatctagtgattggtaacagcggtgatccaaagtacatagatctccctgagatcgaggagaataaaatgtacatagcgaaagaaggttattgttacatcaatatcttcctagccatgttggtaaatgtcaaggagtcgcaggcaaaggagttcacgaaagttgttagggacaaactagttggcgaacttggcaagtggcccactctgttagatgtagcaaccgcttgttatttcctgaaagtattttacccagacgttgctaacgccgaattgccacgcatgctagtggaccataagacaaagataattcatgtcgttgattcatatgggtcactgtcaactggatatcatgtccttaagacaaacactgtggaacaactcatcaaattcacgagatgtaatttggagtcaagcttgaaacactaccgcgttggaggaacagaatgggaggacactcatggatccagcaacatagataatccacagtggtgcatcaagaggctcataaaaggagtctacaaaccaaagcaactgaaagaagacatgttggcaaaccctttcttaccactatatgctctactgtcaccaggtgtcatcctggcattttacaatagtggctctctagagtacttgatgaaccattacatcagggtggacagcaacgtcgccgttttgttggtcgttttgaaatctctagcgaagaaggtgtcaactagtcagagtgtgttagcccagcttcaaatcattgaacgaagtctaccagaactcatcgaagcaaaggctaatgttaatgggccagatgacgcagccactcgcgcgtgtaacagattcatgggcatgcttctgcatatggcagaaccaaactgggagcttgcggatggtggatacacaattctgagggatcatagcatctccattttggaaaaaagttatctacaaatcttggacgaagcatggaacgagttaagttggtcggagcgctgtgctataagatactactcgtcaaagcaagcaatctttacacagaaagatttgccaatgaaaagcgaagccgatttaggcggcagatacagcgtgtcagtcatgtcatcttacgaacggagtaagcaatgtatgaaaagcgtgcactctagtataggtaatagattacgtagtagtatgtcttggactagtagcaaggtgtcgaatagtgtgtgtaggactattaactatttagtaccagatgtgttcaagtttatgaatgtactcgtttgtatcagcttactaatcaagatgactgccgaggcgaatcacatcgtcaccacgcaaagaaggctcaaactagatgtcgaggagacagagcgcaggaaaatagaatgggagcttgcattccaccatgccattctgacgcagagtgcaggtcaacacccaacgatagacgagttcagagcgtacatcgccgacaaggcaccacatctaagtgagcatatcgagcctgaagaaaaggcggtggttcatcaagcgaagagacaatccgagcaagaactcgagcgtataatagcatttgttgcattggtgctcatgatgttcgatgcagaacgaagcgactgtgtcacaaagattctcaacaagcttaagggactagtcgccactgtggaacctacagtctaccatcagactctcaatgatatagaggatgacttgagtgagaggaacctcttcgtcgattttgagcttagcagcgatggagatatgctccaacagcttccagccgaaaagacatttgcctcatggtggagtcatcaactaagcagaggattcacaatcccacactacaggacagaagggaagttcatgactttcaccagagcaactgccacggaagtcgcgggtaaaatagcacacgagagtgacaaagacatattactaatgggagcagtaggatcaggtaagtcaactggcttgccatatcatctctccagaaaagggaacgtattactccttgagccgactcggccacttgcagaaaacgtacacaagcagttgtcgcaggcaccgttccatcagaacacaactcttaggatgcgcggactaacagcattcgggtcggcaccaatctcagtgatgaccagtggttttgcactcaattactttgcaaacaacagaatgcgaattgaagaatttgactttgtcatatttgatgaatgtcacgttcatgacgccaatgcaatggcgatgagatgtttgctacatgagtgtgactattctggcaaaattatcaaagtttcagccacaccaccaggtcgagaagttgagttctccactcaataccccgtgtcgataagcacagaagacacactatcgtttcaggattttgtgaacgcacagggtagtggaagcaattgtgatgtgatttcaaaaggagacaatatcctcgtgtatgtagcaagctacaatgaggtagacgcgctttcaaaacttctaattgaaagagacttcaaagtcacgaaggttgatggaagaacgatgaaagttggaaacatcgagatcaccacaagtggaacacctagtaagaagcacttcatagttgcaaccaacatcatagagaacggtgttactctagacatcgatgtggttgctgattttggaacgaaggtactcccatatcttgatacagacagcagaatgctgagcacaactaagacaagcatcaattatggggaacgtatccaaaggctaggaagagtcggaaggcacaagccaggtcacgctctgcgaataggtcacacagagaaggggttgagcgaagttccaagttgtattgcaacagaagcagctttaaagtgcttcacttatgggcttccagtgatcaccaacaacgtctcgacaagtattcttggtaatgtaacggtaaagcaggcacgaacaatgtctgtatttgagataacaccgttctacacaagccaagtggtgagatatgatggctccatgcatccacaggtgcacgcactcttaaagagattcaaactcagagactctgagattgttttgaataaattagccatacctcaccgaggagtgaacgcttggctcacagctagtgagtatgcacgacttggcgcgaatgttgaagataggcgtgacgttcgaattccttttatgtgtcgcgacatcccagaaaaacttcatctagacatgtgggatgtgattgttaaattcaaaggtgatgcaggttttggtcggctttcaagcgccagtgcgagcaaggtagcttatactctacagacggacgtcaactccatacagcgaacagtcactatcatagatacactaatcgctgaggagagaaggaagcaggaatacttcaagacggtaacctccaactgtgtctcttcttcgaacttctcactgcagagcataacaaatgcgataaaatctcgtatgatgaaagatcacacgtgcgagaacatatcagtgcttgaaggagcgaagtcacagttactcgagtttagaaacctgaatgctgatcactcatttgctacaaaaaccgatggaatatctcggcatttcatgagtgagtatggagctcttgaggcagttcaccatcaaaacaccagcgacatgagcaaattcctcaagcttaagggcaaatggaataaaacgctaatcacgcgagatgtgctggtactttgtggagttcttggaggtggattgtggatggttattcagcacctgcggtcaaagatgtccgaacccgtaacccatgaagcgaaaggtaagaggcaaaggcagaaactaaaatttcgcaatgcccgagacaacaaaatgggtagagaagtgtacggagatgatgataccatagagcatttcttcggtgatgcctacacaaagaaagggaagagcaagggtaggacacgtggtatcggacacaaaaacaggaagttcatcaacatgtatgggtttgatcctgaagatttctctgcagttcgtttcgtggatccactcacaggagcgacgttggacgacaacccgctcacagacatcacccttgtgcaagagcacttcggcaacataagaatggacttactcggggaggatgagctggactcaaatgaaatacgtgtgaataagactattcaagcctactacatgaacaataaaacaggcaaggctttgaaggtggatctgacaccacacatacctctcaaggtgtgtgatcttcacgcaaccattgctggattcccagagcgagaaaacgagctgaggcagactggaaaggctcagcccatcaacatagacgaagtgccaagagctaacaacgaactcgtcccagtggaccacgagagtaactccatgttcagagggttgcgtgactacaacccaatatcaaacaacatttgtcatctcacaaatgtttcagatggagcatcaaactcgttatatggagtcggtttcggaccactcatattaacgaaccgacacctctttgagcggaataacggtgaactcgtaataaaatcacgacatggtgagttcgtgattaaaaacacaactcagctacacttgctaccgattccagacagagatcttctgctaatccggttaccaaaggacgtcccaccctttccacagaaattgggtttcaggcaacctgagaaaggtgaacgaatttgcatggtggggtccaatttccaaaccaagagcataacgagtatagtctctgagactagtacaataatgccagtggagaacagtcagttttggaaacactggattagcactaaagacggccaatgcggaagtccaatggtgagcacgaaagacgggaaaatactcggattacacagcctagcgaacttccagaactccatcaattactttgctgctttcccagatgattttgccgagaagtatcttcataccattgaagcacacgagtgggtcaagcactggaagtataacactagcgccatcagttggggctctttgaatatacaagcatcgcaaccgtccggcttgttcaaagtaagcaagctaatctcagacctcgacagcacggcagtctacgcacaaacccagcagaatcggtggatgttcgagcagctcaacgggaacctaaaagcgatagcacactgccctagccagcttgtgacaaagcacacagttaaaggaaaatgtcagatgtttgacttgtatctcaagttgcatgatgaagcacgagagtatttccaaccgatgctgggccagtatcaaaagagcaaactcaatcgagaagcatatgcaaaggatcttctgaaatatgcaacgccaatcgaagcaggaaacatcgactgtgatctgtttgaaaagacagttgaaatagtcgtatcagatctgcgaggttatggtttcgaaacatgcaattatgtcactgatgagaatgacatattcgaagctcttaacatgaaatccgcagttggagcgttgtataaaggaaagaagaaggattacttcgctgagttcacacccgagatgaaagaagaaatactgaaacaaagttgtgaacggctcttcctaggaaagatgggagtgtggaacggctcgctgaaggcagagttgcgaccactagaaaaagtggaagcaaacaaaacacggacgtttactgccgcaccactagacacactgttgggtggaaaagtttgcgtggatgatttcaacaaccagttctatgatcacaaccttagagctccttggagcgttggcatgacaaagttttattgtggttgggatcgcttgttggagtcgttgccagatggttgggtgtattgcgatgctgatggctcacagttcgacagctcgctatcgccatacttgatcaacgcagtactcaacatccgcttaggattcatggaagagtgggacataggggaggtaatgctgagaaatttgtacaccgaaatcgtgtatacccctatttctacaccagatggtacactcgtcaagaagttcaaaggaaacaatagcggacagccatcgactgttgtggacaacacgctcatggtcatattggcagtcaactattcactcaagaaaagcggaattccaagtgagttgcgcgacagcatcatcagattcttcgtcaacggagatgatttactgctaagcgtacacccagagtatgagtatattcttgacactatggcagacaactttcgtgaactgggcctgaagtatactttcgactcaagaaccagggaaaaaggagacctctggtttatgtcgcaccaggggcacaaaagagagggaatctggattcccaagctcgagccagagcgaatagtatcgattctagaatgggatcggtcgaaagagccatgccatcgactagaggcaatctgcgcagcgatgattgagtcgtggggatacgacaagttaactcacgagatacgcaagttctacgcgtggatgattgaacaagctccatttagctccctagcacaagaagggaaagctccttacatagcggaaacagcgctgaggaagctctaccttgataaggaaccagctcaagaggatctcacccattatttgcaagcaatctttgaggattatgaagatggtgctgaggcttgtgtttatcaccaggcaggtgaaacgcttgatgcaggtttgacagacgagcaaaagcaggcagagaaggagaagaaggagagagagaaggcagaaaaggaacgagagaggcaaaagcagttggcactcaagaaaggcaaggatgttgcacaagaagagggaaaacgcgacaag**gaagtaaacgctggaacctctggaactttcagtgta**cccagactcaagagtctgacaagcaagatgcgcgtgccaagatacgagaaaagagtggctctaaacctcgatcatctaatcctatacacgccggagcagacggatctatccaacacacgttcaacgcgaaagcagtttgacacatggtttgaaggtgtaatggctgattacgaactgacggaggacaaaatgcaaatcattctcaatggtttaatggtctggtgcattgagaacggaacctccccgaacataaacggaatgtgggtgatgatggacggcgacgatcaggtggaattcccgatcaaaccgctcattgaccacgccaaacccacatttaggcagataatggcccatttcagtgacgtagctgaagcgtacattgaaaagcgtaaccaagaccgaccatacatgccacgatatggtcttcagcgcaatttaaccgacatgagcttagctcgatacgcatttgatttctatgaaatgacttctaggactccaatacgtgcgagagaggcacacatccagatgaaagcagcagcactgcgtggcgcaaataataatttgttcggcttggatggaaacgttggtacaacggtagagaacacggagaggcatacgaccgaggacgttaatcggaacatgcataacttactgggcgttcaggggttgtgaagttgtatgctggtagactataagtatttaagtttactcgttagtattctcgcttatgggaaatatgtaagtttgttaaagcagccagtgtgactttgtcatgtgtgttgttgttactttctgtattttcgccgaacattttattggtgttagcgcatgtagtgaggatcgtcctcgattgccttaacatttgataggatgcaagggacaaaaaaaaaaaaaaaaaaaaaaaaaaaaaaaaaaaaaaaaaaaaaaaaaaaaaa


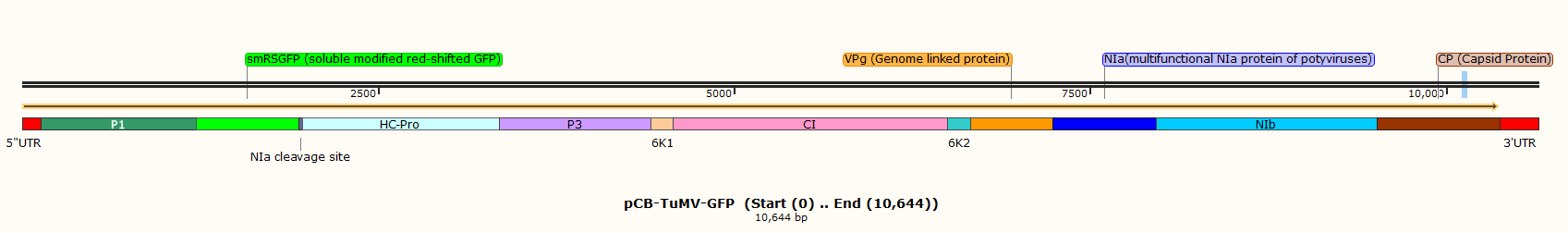
**Supplementary Map 2:** Map of *TuMV-GFP*

**Sequence S4:** Cas13a-repeat-cRNA-TuMV-GFP-GFP-target 1 sequence (A), map (B) and its complex with targeting region in TuMV GFP genomic region (C)

A


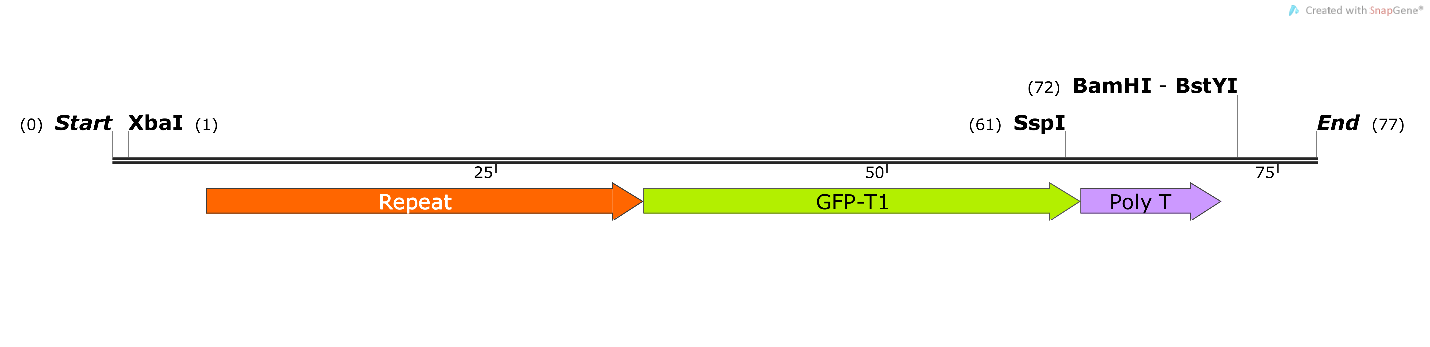
TCTAGA**CCACCCCAATATCGAAGGGGACTAAAACAACAGGTAGTTTTCCAGTAGTGCAAATATTTTTTTTT**GGATCC

B

Cas13a repeat-TuMV-GFP-T1-crRNA for SPDK-TRV (77bp)

UAAAUUUAUUUGCACUACUGGAAAACUACCUGUUCCUUGG

5’

3’

ACC

G

G

G

G

A

A

U

A

U

C

G

A

A

C

C

C

C

CAAAAUCA

AUAAACGUGAUGACCUUUUGAUGGACAA

PFS

Cas13-repeat-GFP-T1-crRNA

GFP Targeting sequence in TuMV

C

**Sequence S5:** Cas13a-repeat-cRNA-TuMV-GFP-GFP-T2 sequence (A), map (B) and its complex with targeting area in TuMV GFP genomic region (C).

A


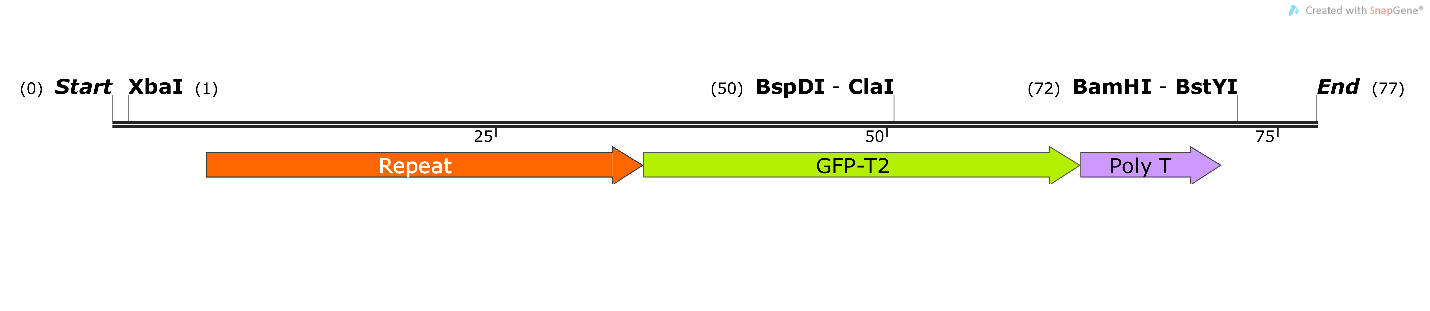
TCTAGA**CCACCCCAATATCGAAGGGGACTAAAACCCGTCCTCCTTGAAATCGATTCCCTTAATTTTTTTTT**GGATCC

B

Cas13a repeat-TuMV-GFP-T2-crRNA for SPDK-TRV (77bp)

C

PFS

ACC

G

G

G

G

A

A

U

A

U

C

G

A

A

C

C

C

C

CAAAAUCA

CGAGCUUAAGGGAAUCGAUUUCAAGGAGGACGGAAACAU

AAUUCCCUUAGCUAAAGUUCCUCCTGCC

5’

3’

Cas13-repeat-GFP-T2-crRNA

GFP Targeting sequence in TuMV

**Sequence S6:** Cas13a-repeat-cRNA-TuMV-GFP-HC-Pro-T1 sequence (A), map (B) and its complex with HC-pro targeting region in TuMV (C).

A


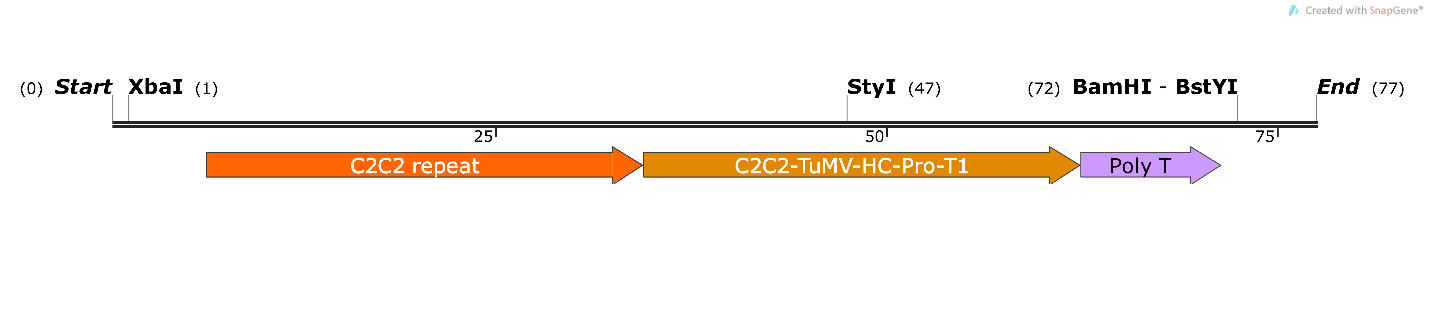
TCTAGA**CCACCCCAATATCGAAGGGGACTAAAACCCGCTTGCTTGTCCTTGGGATAGCTCACTTTTTTTTT**GGATCC

B

Cas13a repeat-TuMV-HC-Pro-T1-crRNA for SPDK-TRV (77bp)

C

ACC

G

G

G

G

A

A

U

A

U

C

G

A

A

C

C

C

C

CAAAAUCA

CAGAUAGUGAGCUAUCCCAAGGACAAGCAAGCGGACCAUCU

CACUCGAUAGGGUUCCUGUUCGUUCGCC

PFS

Cas13-repeat-HC-PRO-crRNA

5’

3’

HC-pro Targeting sequence in TuMV

**Sequence S7:** Cas13a-repeat-cRNA-TuMV-GFP-Cp-Pro-T1 sequence (A), map (B) and its complex with Capsid protein targeting region in TuMV.

A

TCTAGA**CCACCCCAATATCGAAGGGGACTAAAACACACTGAAAGTTCCAGAGGTTCCAGCGTTTTTTTTTT**GGATCC


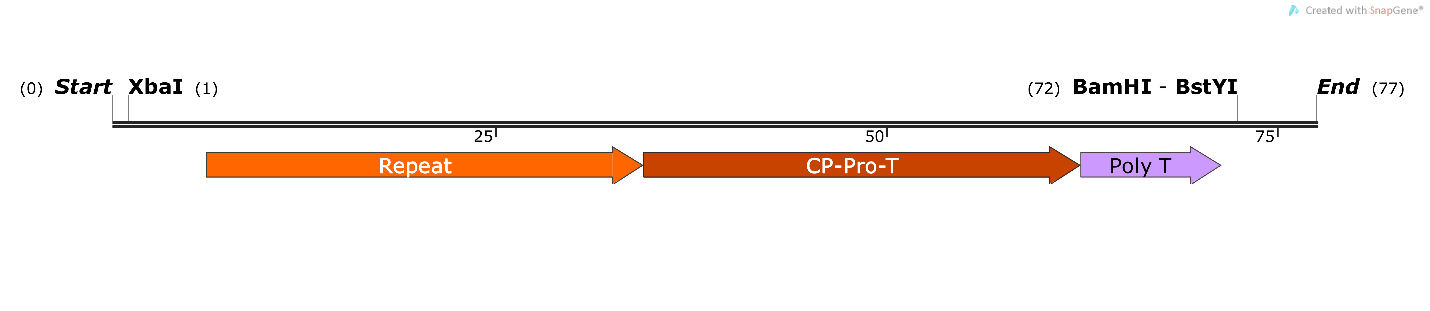


B

Cas13a repeat-TuMV-HC-Pro-T1-crRNA for SPDK-TRV(77bp)

C

PFS

ACC

G

G

G

G

A

A

U

A

U

C

G

A

A

C

C

C

C

CAAAAUCA

UGCGACCUUGGAGACCUUGAAAGUCACA

GAAGUAAACGCUGGAACCUCUGGAACUUUCAGUGUACCCA

5’

3’

Cp-pro Targeting sequence in TuMV

Cas13-repeat-CP-pro-T-crRNA
