# Supplementary material for: RNA virus interference via CRISPR/Cas13a system in plants
Source: Genome Biol. 2018 Jan 4;19:1. doi: 10.1186/s13059-017-1381-1 (PMC5755456; doi:10.1186/s13059-017-1381-1)
Supplement: Supplementary file 3 — TuMV genome description. Table S2: Primers used in this study. (DOCX 21 kb) [file 13059_2017_1381_MOESM3_ESM.docx]

**ADDITIONAL FILE 3**

**RNA virus interference via CRISPR/Cas13a system in plants**

Rashid Aman^1, 3^, Zahir Ali^1, 3^, Haroon Butt^1, 3^, Ahmed Mahas^1^, Fatimah Aljedaani^1^, Muhammad

Zuhaib Khan^1^, Shouwei Ding^2^, and Magdy Mahfouz^1, *^

^1^Laboratory for Genome Engineering, Division of Biological Sciences, 4700 King Abdullah University of Science and Technology, Thuwal 23955-6900, Saudi Arabia, and ^2^Center for Plant Cell Biology, Department of Microbiology and Plant Pathology, University of California, Riverside, CA 92521

***Corresponding author:** Magdy M. Mahfouz ([magdy.mahfouz@kaust.edu.sa)](mailto:magdy.mahfouz@kaust.edu.sa))

**Additional file 3.**

**Table S1: TuMV genome description**

**Table S2: Primers used in this study**

**Table S1: TuMV genome description**

| **Name** | **Size** | **Full name and function** |
| --- | --- | --- |
| 5′ UTR | 1–130 bp (130 bp) | 5′ untranslated region |
| P1 | 131–1219 (1089 bp) | The P1 protein is a multifunctional protein involved in cell to cell movement, systemic spread and viral genome replication enhancement. |
| GFP | 1220–1939 (720 bp) | Green fluorescent protein for detection of virus under UV light or Western blot |
| Nia | 1940–969 (30 bp) | Nuclear inclusion protein a |
| Hc-Pro | 1970–3346 (1377 bp) | Helper component proteinase silencing suppressor |
| P3 | 3347–4411 (1065 bp) | the P3 protein |
| 6K1 | 4412–4567 (156 bp) | First peptide of 6 kDa |
| CI | 4568–6499 (1932 bp) | Cylindrical inclusion |
| 6K2 | 6500–6658 (159 bp) | Second peptide of 6KDa |
| VPg | 6659–7234 (576 bp) | viral genome linked protein helps in systemic infection |
| Nia | 7235–7963 (729 bp) | Nuclear inclusion protein a |
| Nib | 7964–9514 (1551 bp) | Nuclear inclusion protein b |
| CP | 9515–10378 (864 bp) | Coat / Capsid protein |
| 3′ UTR | 10382–10644 (263 bp) | 3′ untranslated region |

**Table S2: Primers used in this study**

| **Primer name** | **Sequence (5′ to 3′)** | **Usage** |
| --- | --- | --- |
| Cas13a-TuMV-GFP-T1-TRV-F | CTAGACCACCCCAATATCGAAGGGGACTAAAACAACAGGTAGTTTTCCAGTAGTGCAAATATTTTTTTTTG | Forward primer for cloning of Cas13a-repeat-crRNA-GFP-T1 under PEBV promoter in TRV system |
| Cas13a-TuMV-GFP-T1-TRV-R | GATCCAAAAAAAAATATTTGCACTACTGGAAAACTACCTGTTGTTTTAGTCCCCTTCGATATTGGGGTGGT | Reverse primer for cloning of Cas13a-repeat-crRNA-GFP-T1 under PEBV promoter in TRV system |
| Cas13a-TuMV-GFP-T2-TRV-F | CTAGACCACCCCAATATCGAAGGGGACTAAAACCCGTCCTCCTTGAAATCGATTCCCTTAATTTTTTTTTG | Forward primer for cloning of Cas13a-repeat-crRNA-GFP-T2 under PEBV promoter in TRV system |
| Cas13a-TuMV-GFP-T2-TRV-R | GATCCAAAAAAAAATTAAGGGAATCGATTTCAAGGAGGACGGGTTTTAGTCCCCTTCGATATTGGGGTGGT | Reverse primer for cloning of Cas13a-repeat-crRNA-GFP-T1 under PEBV promoter in TRV system |
| Cas13a-TuMV-HC-Pro-T1-TRV-F | CTAGACCACCCCAATATCGAAGGGGACTAAAACCCGCTTGCTTGTCCTTGGGATAGCTCACTTTTTTTTTG | Forward primer for cloning of Cas13a-repeat-crRNA-HC-Pro-T1 under PEBV promoter in TRV system |
| Cas13a-TuMV-HC-Pro-T1-TRV-R | GATCCAAAAAAAAAGTGAGCTATCCCAAGGACAAGCAAGCGGGTTTTAGTCCCCTTCGATATTGGGGTGGT | Reverse primer for cloning of Cas13a-repeat-crRNA-HC-Pro-T1 under PEBV promoter in TRV system |
| Cas13a-TuMV-Cp-Pro-T1-TRV-F | CTAGACCACCCCAATATCGAAGGGGACTAAAACACACTGAAAGTTCCAGAGGTTCCAGCGTTTTTTTTTTG | Forward primer for cloning of Cas13a-repeat-crRNA-Cp-Pro-T1 under PEBV promoter in TRV system |
| Cas13a-TuMV-Cp-Pro-T1-TRV-R | GATCCAAAAAAAAAACGCTGGAACCTCTGGAACTTTCAGTGTGTTTTAGTCCCCTTCGATATTGGGGTGGT | Reverse primer for cloning of Cas13a-repeat-crRNA-Cp-Pro-T1 under PEBV promoter in TRV system |
| Cas13a-TRV-ns-crRNA-T1-F | CTAGACCACCCCAATATCGAAGGGGACTAAAACTCCGGATCCAGAGAGATGATTCTCCCGCTTTTTTTTTG | Forward primer for cloning of Cas13a-repeat-crRNA-Nonspecific-T1 under PEBV promoter in TRV system |
| Cas13a-TRV-ns-crRNA-T1-R | GATCCAAAAAAAAAGCGGGAGAATCATCTCTCTGGATCCGGAGTTTTAGTCCCCTTCGATATTGGGGTGGT | Forward primer for cloning of Cas13a-repeat-crRNA-Nonspecific-T1 under PEBV promoter in TRV system |
| \| pCas13a- repeat \| GUUUUAGUCCCCUUCGAUAUUGGGGUGG \| \| --- \| --- \| | GUUUUAGUCCCCUUCGAUAUUGGGGUGG | Synthetic dig labelled probe |
| T7-TuMV-F | \| TAATACGACTCACTATAGGCATATGAAGCGGCACGACTTCTTCAAGAGCGCC \| TAATACGACTCACTATAGGcatatgaagcggcacgacttcttcaagagcgcc \| \| --- \| --- \| \|  \| TAATACGACTCACTATAGGcatatgaagcggcacgacttcttcaagagcgcc \| | For making dig labelled probe for detection of TuMV-GFP genome |
| TuMV-RT-R | \| CCATTCTTTTGTTTGTCTGCCGTGA \| ccattcttttgtttgtctgccgtga \| \| --- \| --- \| \|  \| ccattcttttgtttgtctgccgtga \| | For making dig labelled probe for detection of TuMV-GFP genome |
| crRNA-Sequence | CCACCCCAAUAUCGAAGGGGACUAAAACUUUGCUCCCCUCCACAAGAACAUUGAGA | Synthetic crRNA positive control |
